# Overall Survival Benefit in Rectal Cancer After Neoadjuvant Radiotherapy and Adjuvant Chemotherapy: A Propensity-Matched Population-Based Study

**DOI:** 10.3389/fonc.2020.584835

**Published:** 2020-12-09

**Authors:** Zhiju Chen, Shaowei Li, Yehong Wang, Zhiming Fu, Ning Liu, Hao Wang, Xin Liu

**Affiliations:** ^1^ The First Department of Gastrointestinal Surgery, Hainan General Hospital, Hainan Affiliated Hospital of Hainan Medical University, Haikou, China; ^2^ Department of Oncology, Nanfang Hospital, Southern Medical University, Guangzhou, China

**Keywords:** rectal cancer, neoadjuvant radiotherapy, chemotherapy, survival, propensity score match

## Abstract

**Background:**

It is well known that neoadjuvant radiotherapy could reduce local recurrence followed by surgical resection. However, evidence about oncologic efficacy of radiotherapy and survival benefit of adjuvant chemotherapy after neoadjuvant radiotherapy is still lacking.

**Methods:**

This retrospective propensity score-matched cohort study identified patients with pathologically confirmed rectal cancer and receiving surgery with curative intent from the Surveillance, Epidemiology, and End Results database from 2004 through 2014. Overall survival was compared using the stratified log-rank test. Multivariate Cox regression analysis was used for identifying risk factor and developing prediction nomogram.

**Results:**

A total of 22,008 (11,004 for each group) propensity-matched patients were identified. In the context of receiving adjuvant chemotherapy after surgical resection, there was no significant difference in terms of overall survival between surgery alone group and neoadjuvant radiotherapy and surgery group, whether for stage I (log-rank test *p* = 0.467), stage II (log-rank test *p* = 0.310), or stage III (*p* = 0.994). In case of receiving a prior combination therapy of neoadjuvant radiotherapy and surgery, the following adjuvant chemotherapy could significantly improve overall survival for patients with stage I (log-rank test *p <*0.001), stage II (log-rank test *p* = 0.038), and stage III (log-rank test *p* = 0.014). Nomogram integrating clinicopathologic factors was developed to predict survival benefit associated with neoadjuvant radiotherapy. Calibration and ROC curves validated promising performance for the nomogram.

**Conclusion:**

Patients with rectal cancer underwent neoadjuvant radiotherapy yield acceptable outcomes and are more likely to benefit from adjuvant chemotherapy in terms of overall survival. These data would be evidential for advocating consistency in guideline adherence to the use of adjuvant chemotherapy after neoadjuvant radiotherapy.

## Introduction

The role of neoadjuvant radiotherapy in the treatment of rectal cancer has been established in the last few decades (1). It is well known that neoadjuvant radiotherapy could reduce local recurrence and enhance survival followed by surgical resection (2). Current recommendations for treatment of patients with locally advanced rectal cancer include preoperative neoadjuvant radiotherapy followed by surgical resection and adjuvant chemotherapy, irrespective of pathological stage (3).

Benefits of patients with rectal cancer from additional neoadjuvant radiotherapy is growing with the continuous improvement of neoadjuvant regimen. Downstaging after neoadjuvant therapy is known as a prognostic factor for rectal cancer and it also has been demonstrated that patients survive in a manner that most closely associates with their tumor stage after neoadjuvant therapy and differs far from their pre-treatment stage (4–7). Although the use of neoadjuvant radiotherapy has been supported by favorable prognosis for patients over those with relative advanced stage before neoadjuvant therapy, currently, two major concerns about neoadjuvant radiotherapy for rectal cancer from clinical setting are still unsettled. One is the oncologic efficacy of the neoadjuvant radiotherapy; could patients yield equivalent even superior survival outcomes after neoadjuvant radiotherapy than those stage-matched patients without neoadjuvant therapy? Another is about the management strategy after neoadjuvant therapy and surgical resection, which is mainly focused on the application of adjuvant chemotherapy for such patients (8–10). Estimates of survival benefit with neoadjuvant radiotherapy and adjuvant chemotherapy in patients with advanced rectal cancer are various, as suggested by recently reports and clinical trials (11–17).

To address above issues, through querying a large population-based cancer database for patients with rectal cancer who underwent surgical resection over a 10-year time period, this study seeks to evaluate the oncologic efficacy of neoadjuvant radiotherapy and to determine whether adjuvant chemotherapy is associated with improved overall survival in patients after neoadjuvant radiotherapy.

## Methods

### Database and Patient Population

**Figure 1:**
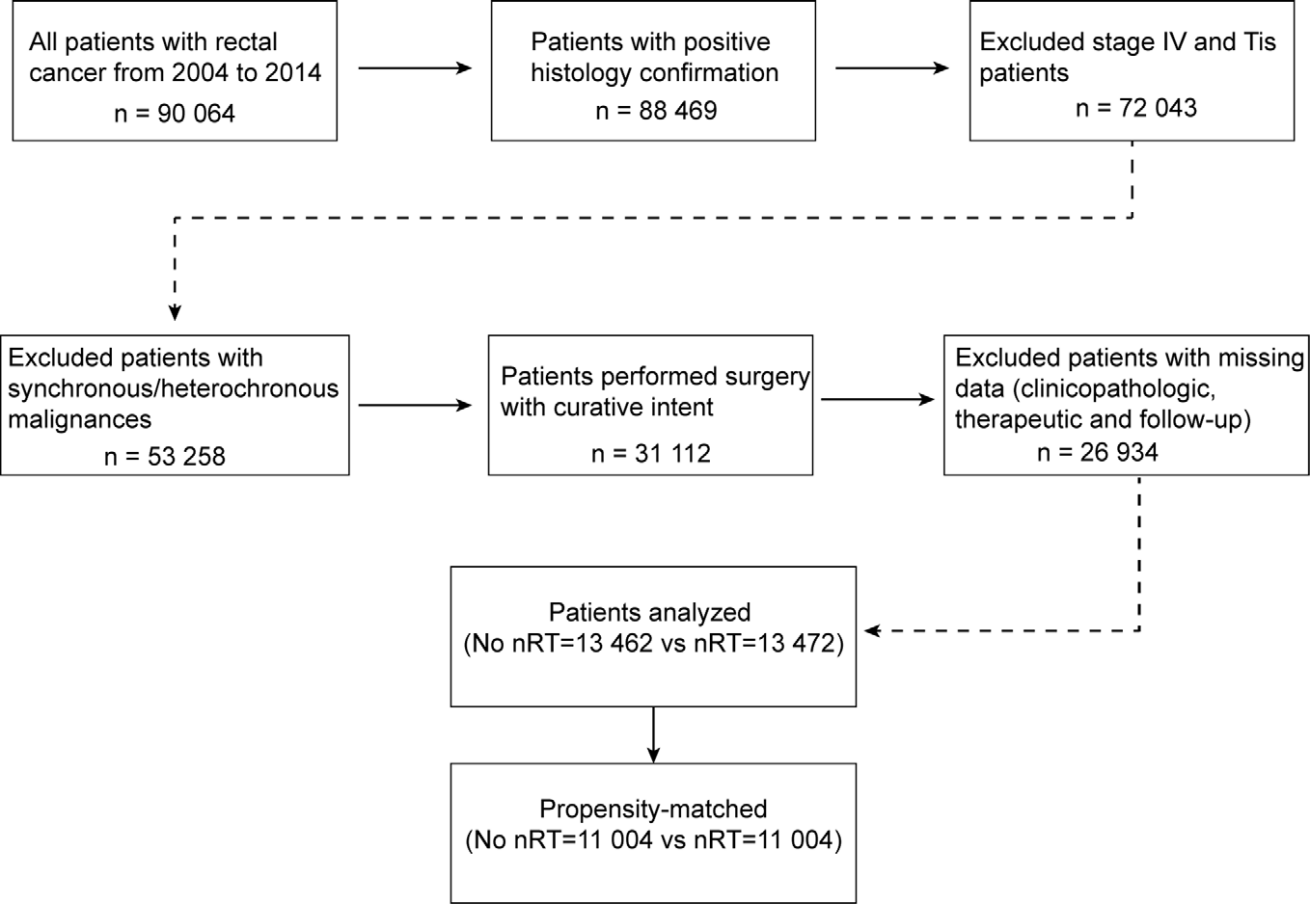
Inclusion algorithm. nRT indicates receipt of neoadjuvant radiotherapy.

The Surveillance, Epidemiology, and End Results (SEER) Program of the National Cancer Institute (NCI) is one of biggest population-based cancer registries, collecting cancer incidence, prevalence and survival data covering approximately 34.6% of the US population (18). Access to the de-identified linked dataset was obtained after SEER approval of a custom data request and signature of a Data-Use Agreement. For analyses of de-identified data from the SEER registry, local institutional review board approval and informed consent were not required. Anonymized, patient-level data were extracted from the publicly available online SEER database for 18 defined geographic regions across the United States. This study cohort consisted of all patients with resected rectal cancer diagnosed between 2004 and 2014. For this analysis, the April 2017 release of the SEER 18 database was used for case extraction. Initial patient selection was based on the SEER Site Recode “rectum“ (equivalent to International Classification of Disease for Oncology, 3th edition site code C209). Patients with positive histology confirmation and receiving cancer-directed surgery with curative intent were included. Patients with stage Tis or stage IV disease, and synchronous/heterochronous malignances, and those with missing clinicopathologic, therapeutic or follow-up information, were excluded from this analysis. A detailed inclusion algorithm is shown in **Figure 1**. This study was approved by the appropriate institutional review board. Anonymized data were used for analyses, and therefore no consents were required.

### Survival Analysis and Propensity Score Matching

The primary outcome of interest in present study was overall survival (OS), which was measured from the time of diagnosis. Patient characteristics examined included age at diagnosis, sex, race, tumor grade, histology classification, receipt of neoadjuvant radiotherapy and adjuvant chemotherapy, and tumor stage. Unbalanced distribution of patient’s characteristics lying in a raw database-derived population from multiple clinic units, often make difficulties to produce a statistical conclusion. To adjust for such bias, propensity score matching was proposed to control the bias from those covariates influencing the treatment-selection process (19, 20). In this analysis, those important prognostic factors in combination with availability of records in SEER, including age, sex, race, histology classification, and differentiation grade, were used as covariates for propensity score modeling. The propensity score was defined as the probability of patients receiving neoadjuvant radiotherapy, estimated by a non-parsimonious multivariable logistic regression model. A nearest neighbor matching algorithm (ratio = 1:1 without replacement) was performed with a caliper of width 0.2 standard deviations of the logit model of the estimated score.

### Multivariate Regression Analysis and Nomogram Construction

Multivariate regression analysis was performed by using the Cox proportional hazard model. Continuous variable was modeled using a restricted spline function with independent coefficients; the interaction between tumor stage and the receipt of neoadjuvant radiotherapy was entered into the model to reflect the impact from the tumor stage on the survival benefits of neoadjuvant radiotherapy. The coefficients from the multivariate Cox regression analysis were used for nomogram construction to predict the survival outcomes. Two dimensions of calibration and discrimination were used to validate the model performance, by using the calibration curve and the receiver operating characteristic (ROC) curve, respectively.

### Statistical Analysis

Categorical variables were presented as frequency (percentage) and compared using chi-square test, and continuous data were presented as mean (standard deviation) and compared using two independent samples *t* test. Survival curve was estimated using Kaplan-Meier methodology and compared by log-rank test. Hazard ratio (HR) was estimated by using Cox proportional hazard regression model. SEER data were extracted by using Client-Server Mode with SEER*Stat 8.3.5. Statistical analyses were performed by using SPSS Statistics version 24 (IBM, Chicago, IL), and R software (https://www.r-project.org/) and its optional packages. A two-tailed *p* value <0.05 was considered statistically significant.

## Results

### Patient Population and Propensity Score Matching

This study finally included a total of 26,934 patients for analysis, including 13,462 patients undergoing surgical resection up front and 13,472 patients receiving neoadjuvant radiotherapy prior to surgical resection. Baseline clinicopathologic characteristics are detailed in **Table 1**. Patients receiving neoadjuvant radiotherapy tended to be younger (*p* < 0.001) and male (*p <*0.001). There existed systematic differences in clinicopathologic data between the two groups in overall samples. Therefore, in order to balance the baseline characteristics, a propensity score matching (ratio = 1:1) was performed by using five covariates with unbalanced distribution (i.e., age, sex, race, histology classification, and grade). After matching, 11,004 patients were selected in each group, with no significant difference among above covariates, indicating a balanced baseline between the two groups (**Table 2**).

**Table 1:** Patient baseline characteristics before matching.

	No neoadjuvant radiotherapy (n=13462) N (%) or mean (SD)	Neoadjuvant radiotherapy (n=13472) N (%) or mean (SD)	*p*
Age (year)	63.65 (13.55)	58.91 (12.31)	<0.001
Sex (male)	7591 (55.3)	8510 (62.8)	<0.001
Race			<0.001
White	11282 (82.2)	11004 (81.2)	
Asian/Pacific Islander	1334 (9.7)	1314 (9.7)	
American Indian/Alaska Native	76 (0.6)	129 (1.0)	
Black	1033 (7.5)	1099 (8.1)	
Histology classification (ICD-O-3)			<0.001
Adenocarcinomas (8140-8320)	12680 (94.2)	12400 (92.0)	
Mucinous and serous (8480–8490)	704 (5.2)	970 (7.2)	
Others	78 (0.6)	102 (0.8)	
Grade			<0.001
Grade I (well differentiated)	1233 (9.2)	988 (7.3)	
Grade II (moderately differentiated)	10264 (76.2)	10500 (77.9)	
Grade III (poorly differentiated)	1775 (13.2)	1817 (13.5)	
Grade IV (undifferentiated)	190 (1.4)	167 (1.2)	
Adjuvant chemotherapy	5137 (38.2)	13130 (97.5)	<0.001
Tumor depth (T stage)			<0.001
T1	3190 (23.7)	894 (6.6)	
T2	3562 (26.5)	1963 (14.6)	
T3	6032 (44.8)	9498 (70.5)	
T4	678 (5.0)	1117 (8.3)	
Lymph node invasion (N stage)			<0.001
N0	8504 (63.2)	6761 (50.2)	
N1	3086 (22.9)	5085 (37.7)	
N2	1872 (13.9)	1626 (12.1)	
TNM stage			<0.001
I	5427 (40.3)	1994 (14.8)	
IIA	2795 (20.8)	4277 (31.7)	
IIB	281 (2.1)	489 (3.6)	
IIIA	1061 (7.9)	715 (5.3)	
IIIB	2026 (15.0)	4371 (32.4)	
IIIC	1872 (13.9)	1626 (12.1)	

TNM staging was performed according to American Joint Committee on Cancer (AJCC), and was converted to 6th edition accordingly for all patients. SD, standard deviation; ICD-O-3, International Classification of Disease for Oncology, 3th edition.

**Table 2:** Patient baseline characteristics after matching.

	No neoadjuvant radiotherapy (n=11004) N (%) or mean (SD)	Neoadjuvant radiotherapy (n=11004) N (%) or mean (SD)	*p*
Age (year)	60.53 (11.88)	60.70 (11.96)	0.293
Sex (male)	6603 (60.0)	6603 (60.0)	>0.999
Race			>0.999
White	9071 (82.4)	9071 (82.4)	
Asian/Pacific Islander	833 (7.6)	833 (7.6)	
American Indian/Alaska Native	1046 (9.5)	1046 (9.5)	
Black	54 (0.5)	54 (0.5)	
Histology classification (ICD-O-3)			>0.999
Adenocarcinomas (8140-8320)	10389 (94.4)	10389 (94.4)	
Mucinous and serous (8480-8490)	574 (5.2)	574 (5.2)	
Others	41 (0.4)	41 (0.4)	
Grade			>0.999
Grade I (well differentiated)	884 (8.0)	884 (8.0)	
Grade II (moderately differentiated)	8657 (78.7)	8657 (78.7)	
Grade III (poorly differentiated)	1359 (12.4)	1359 (12.4)	
Grade IV (undifferentiated)	104 (0.9)	104 (0.9)	
Adjuvant chemotherapy	4540 (41.3)	10705 (97.3)	<0.001
Tumor depth (T stage)			<0.001
T1	2757 (25.1)	762 (6.9)	
T2	2898 (26.3)	1639 (14.9)	
T3	4829 (43.9)	7722 (70.2)	
T4	520 (4.7)	881 (8.0)	
Lymph node invasion (N stage)			<0.001
N0	6975 (63.4)	5673 (51.6)	
N1	2493 (22.7)	4078 (37.1)	
N2	1536 (14.0)	1253 (11.4)	
TNM stage			<0.001
I	4555 (41.4)	1710 (15.5)	
IIA	2210 (20.1)	3561 (32.4)	
IIB	209 (1.9)	401 (3.6)	
IIIA	879 (8.0)	581 (5.3)	
IIIB	1615 (14.7)	3498 (31.8)	
IIIC	1536 (14.0)	1253 (11.4)	

TNM staging was performed according to American Joint Committee on Cancer (AJCC), and was converted to 6th edition accordingly for all patients. SD, standard deviation; ICD-O-3, International Classification of Disease for Oncology, 3th edition.

### Survival Outcome for Patients Receiving Neoadjuvant Radiotherapy

**Figure 2:**
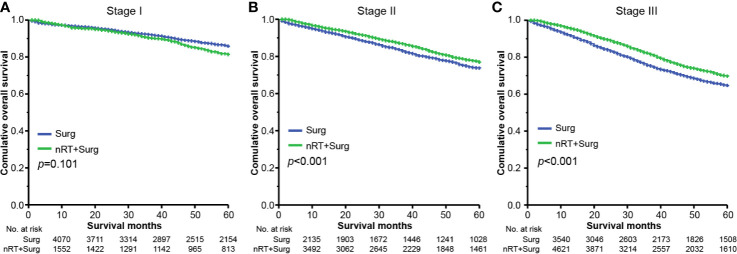
Overall survival for stage I-III rectal cancer. **(A)** Kaplan-Meier plots for overall survival comparison between patients undergoing up-front resection and patients who received neoadjuvant radiotherapy preoperatively in stage I; **(B)** Overall survival plots for patients with stage II; **(C)** Overall survival plots for patients with stage III. “Surg” indicates patients proceeded directly to surgical resection, and “nRT + Surg” indicates patients who received neoadjuvant radiotherapy and surgical resection.

To evaluate the survival benefit from neoadjuvant radiotherapy, the present analysis compared survival outcomes of patients receiving neoadjuvant radiotherapy prior to surgical resection with stage-matched patients who underwent surgery without neoadjuvant therapy. Comparisons were performed within the propensity-matched cohorts. In stage I tumors, compared to patients without neoadjuvant radiotherapy, the improved OS in those receiving neoadjuvant therapy were not observed [mean survival time, with neoadjuvant radiotherapy 55.9 (95% CI, 55.3–56.6) months vs. without neoadjuvant radiotherapy 56.8 (95% CI, 56.4–57.2) months; HR = 1.301 (95% CI, 0.912–1.516); *p* = 0.101] (**Figure 2A**). However, in stage II tumors, the improved OS was observed among patients receiving neoadjuvant radiotherapy (mean survival time 54.3 months, 95% CI: 53.8–54.8 months), as compared with patients without neoadjuvant therapy [mean survival time 52.6 months, 95% CI: 51.9–53.3 months; HR = 0.820 (95% CI, 0.740–0.908); *p <*0.001] (**Figure 2B**). Similar improved outcomes were also found in patients with stage III rectal cancer [mean survival time, with neoadjuvant radiotherapy 52.1 (95% CI, 51.7–52.6) months vs. without neoadjuvant radiotherapy 49.2 (95% CI, 48.6–49.8) months; HR = 0.792 (95% CI, 0.735–0.853); *p <*0.001] (**Figure 2C**).

### Survival Benefit for Patients Receiving Adjuvant Chemotherapy After Neoadjuvant Radiotherapy

**Figure 3:**
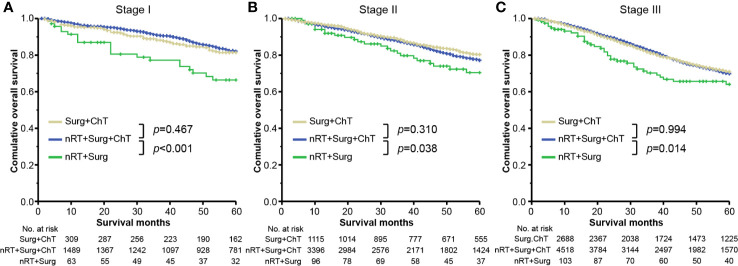
Overall survival plots stratified by therapy combinations for rectal cancer in stage **(A)** I, **(B)** II, and **(C)** III. “Surg + ChT”, patients proceeded directly to surgical resection and receiving postoperatively adjuvant chemotherapy; “nRT + Surg + ChT”, patients receiving combination therapy of neoadjuvant radiotherapy, surgical resection and adjuvant chemotherapy; “nRT + Surg”, patients receiving neoadjuvant radiotherapy and surgical resection, without adjuvant chemotherapy.

It is worth mentioning that 10,705 (97.3%) patients undergoing neoadjuvant radiotherapy also received adjuvant chemotherapy. We further performed an exploratory analysis by considering chemotherapy as a separate variable, and compared patients with full therapy of both neoadjuvant radiotherapy and adjuvant chemotherapy, against with those without neoadjuvant radiotherapy or adjuvant chemotherapy (**Figure 3**). In the context of receiving adjuvant chemotherapy after surgical resection, there was no significant difference regarding OS between the group receiving directly surgical resection (marked as “Surg + ChT” group) and the group with neoadjuvant radiotherapy prior to surgical resection (marked as “nRT + Surg + ChT” group), irrespective of tumor stages: stage I [mean survival time, Surg + ChT group 55.0 (95% CI, 53.4–56.6) months vs. nRT + Surg + ChT group 56.2 (95% CI, 55.6–56.9) months; HR = 1.118 (95% CI, 0.827–1.512); *p* = 0.467] (**Figure 3A**), stage II [mean survival time, Surg + ChT group 55.1 (95% CI, 54.3–55.9) months vs. nRT + Surg + ChT group 54.4 (95% CI, 53.9–54.9) months; HR = 0.932 (95% CI, 0.812–1.070); *p* = 0.310] (**Figure 3B**), or stage III [mean survival time, Surg + ChT group 52.1 (95% CI, 51.4–52.7) months vs. nRT + Surg + ChT group 52.3 (95% CI, 51.7–52.7) months; HR = 1.000 (95% CI, 0.918–1.089); *p* = 0.994] (**Figure 3C**). These results supported the promising efficacy of neoadjuvant radiotherapy for stage I-III rectal cancer, when adjuvant chemotherapy is prescribed after surgical resection.

**Figure 4:**
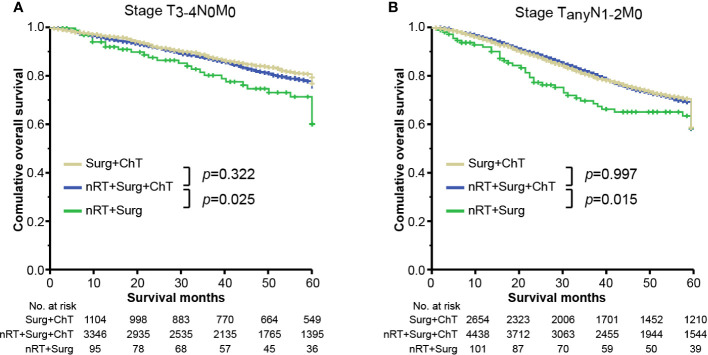
Overall survival plots stratified by therapy combinations for rectal cancer in stage **(A)** T_3-4_N_0_M_0_ and **(B)** T_any_N_1-2_M_0_. “Surg + ChT”, patients proceeded directly to surgical resection and receiving postoperatively adjuvant chemotherapy; “nRT + Surg + ChT”, patients receiving combination therapy of neoadjuvant radiotherapy, surgical resection and adjuvant chemotherapy; “nRT + Surg”, patients receiving neoadjuvant radiotherapy and surgical resection, without adjuvant chemotherapy.

However, in case of receiving a prior combination therapy of neoadjuvant radiotherapy and surgical resection, patients without adjuvant chemotherapy (marked as “nRT + Surg” group) yield worse overall survivals than those with adjuvant chemotherapy (i.e., “nRT + Surg + ChT” group), irrespective of tumor stage. The mean survival time of nRT + Surg group was 49.1 months (95% CI 44.5–53.8; compared with nRT + Surg + ChT group: HR = 2.278, 95% CI 1.457–3.563; *p <*0.001) in stage I tumor (**Figure 3A**), 51.5 months (95% CI 47.9–55.0; compared with nRT + Surg + ChT group: HR = 1.483, 95% CI 1.042–2.019; *p* = 0.038) in stage II tumor (**Figure 3B**), and 47.5 months (95% CI 43.6–51.3; compared with nRT + Surg + ChT group: HR = 1.255, 95% CI 1.011–1.726; *p* = 0.014) in stage III tumor (**Figure 3C**), respectively. We further subgroup analyses for patients with T_3-4_N_0_M_0_ or node-positive rectal cancer. The results show similarly that patients with T_3-4_N_0_M_0_ or node-positive rectal cancer receiving neoadjuvant radiotherapy yielded better survival outcomes after adjuvant chemotherapy than those without postoperative therapy (**Figure 4**). These findings indicated that postoperative chemotherapy was associated with improved survival outcomes of stage I-III rectal cancer after neoadjuvant radiotherapy and surgical resection.

### Multivariate Regression Analysis and Nomogram for Survival Benefit Prediction

**Figure 5:**
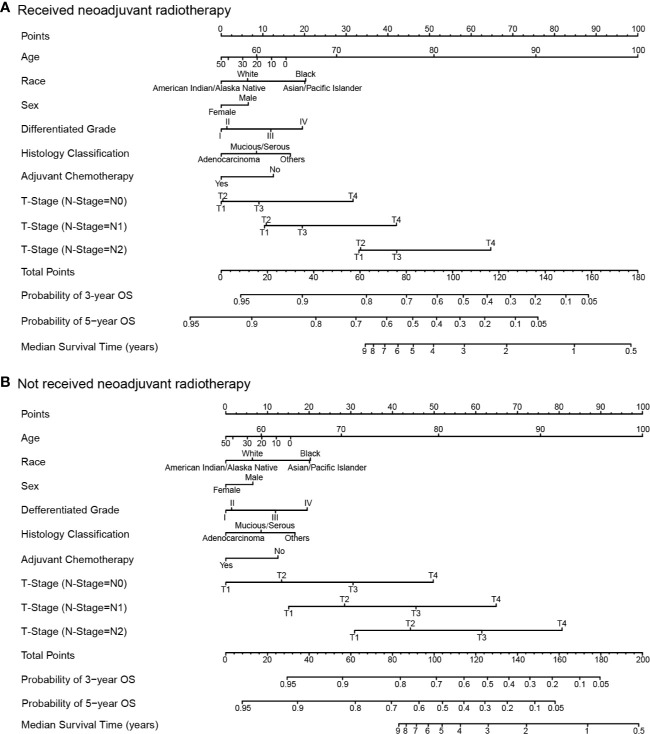
Nomograms for comparing the expected overall survival with and without neoadjuvant radiotherapy. Nomogram **(A)** estimates the expected overall survival with neoadjuvant radiotherapy, and nomogram **(B)** estimates the expected overall survival without neoadjuvant radiotherapy.

Results of the multivariate regression model are listed in **Table 3**. Statistically significant covariates included age, sex, race, histology classification, grade, tumor stage, receipt of adjuvant chemotherapy, and neoadjuvant radiotherapy. As could be seen from the hazard ratios of the interaction terms, the influence of neoadjuvant radiotherapy on survival varied by stages. Nomograms were constructed with the β coefficients from this model. To estimate the net survival benefit from neoadjuvant radiotherapy, the two nomograms were used together (**Figure 5**). The first nomogram (**Figure 5A**) estimated the predicted survival with neoadjuvant radiotherapy, and the second nomogram (**Figure 5B**) estimated survival without neoadjuvant radiotherapy. The difference between the two estimates was the expected net survival benefit from neoadjuvant radiotherapy.

**Table 3:** Cox proportional hazards multivariate regression analysis.

Covariate	β coefficients	Hazard ratio	95% CI	*p*
Age	-0.0094	–†	–	<0.016
Age′	0.0888	–†	–	<0.001
Age″	-0.1708	–†	–	<0.001
Male	0.1760	1.19	1.14–1.25	<0.001
Race (ref. White)				
Asian/Pacific Islander	0.3717	1.45	1.34–1.57	<0.001
American Indian/Alaska Native	-0.1720	0.84	0.77–0.92	<0.001
Black	0.3770	1.46	1.12–1.89	0.004
Histology (ref. Adenocarcinomas)				
Mucinous and serous	0.2295	1.26	1.16–1.37	<0.001
Others	0.4497	1.57	1.24–1.97	<0.001
Grade (ref. Grade I)				
Grade II	0.0375	1.04	0.94–1.14	0.4392
Grade III	0.3229	1.38	1.24–1.54	<0.001
Grade IV	0.5283	1.70	1.40–2.06	<0.001
Adjuvant chemotherapy	-0.3390	0.71	0.66–0.77	<0.001
T stage (ref. T1)				
T2	0.3627	1.44	1.28–1.61	<0.001
T3	0.8272	2.29	2.05–2.55	<0.001
T4	1.3483	3.85	3.32–4.46	<0.001
N stage (ref. N0)				
N1	0.4089	1.51	1.39–1.63	<0.001
N2	0.8370	2.31	2.12–2.52	<0.001
Receiving NRT	0.5793	1.78	1.48–2.16	<0.001
Interaction terms				
T2 × NRT	-0.3737	0.69	0.56–0.85	<0.001
T3 × NRT	-0.5926	0.55	0.46–0.67	<0.001
T4 × NRT	-0.5020	0.61	0.48–0.76	<0.001
N1 × NRT	-0.1272	0.88	0.79–0.99	0.028
N2 × NRT	0.0582	1.06	0.93–1.21	0.386

^†^Age was modeled using a restricted cubic spline function with three independent coefficients, annotated as Age, Age′, and Age″, which yields an effective hazard ratio that varies continuously with age. NRT, neoadjuvant radiotherapy; CI, confidence interval; ref., reference.

**Figure 6:**
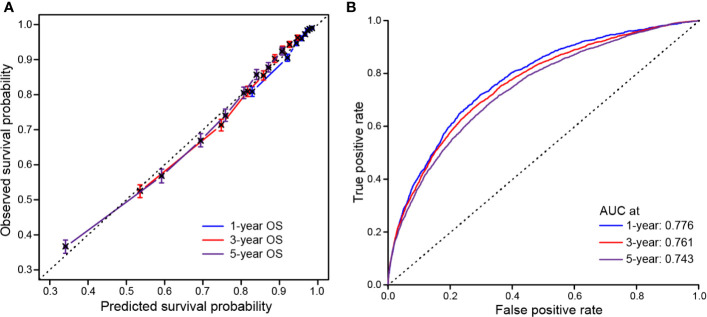
Calibration and discrimination validation. **(A)** Calibration curve demonstrates how survival prediction from the model compare to the actual observed survival; **(B)** Receiver operating characteristic (ROC) curve internally validates the model performance for discrimination.

Model performance was internally validated for calibration and discrimination. The calibration curve (**Figure 6A**) showed good agreement between predicted and observed survival outcomes. Discrimination, as measured by the area under the ROC curve (AUC), was 0.776, 0.761 and 0.743 for 1-, 3-, and 5-year OS, respectively (**Figure 6B**).

## Discussion

Using a large database such as SEER and evaluating more 20,000 patients with rectal cancer over a 10-year period, our findings help to address an important clinical issue. We showed that rectal cancer patients of stage I-III with neoadjuvant radiotherapy yielded acceptable survival outcomes over those undergoing directly surgical resection with final stage-matched disease, and provided evidence to support the survival benefit with adjuvant chemotherapy after neoadjuvant radiotherapy and surgical resection. To date, this represents one of the largest studies analyzing survival in rectal cancer with neoadjuvant radiotherapy, demonstrating overall survival benefits associated with the management strategy of adjuvant chemotherapy after neoadjuvant therapy and surgical resection in rectal cancer.

Given the potential toxicity, adverse effects and associated cost of systematic chemotherapy, it is important to assess the actual benefit of adjuvant chemotherapy. However, the survival benefits associated with adjuvant chemotherapy in patients with rectal cancer after neoadjuvant radiotherapy and surgical resection remains controversial and conclusions varied among previous studies. Several recent reports and clinical trials demonstrated no additional survival benefit with chemotherapy in patients with locally advanced rectal cancer receiving neoadjuvant chemoradiotherapy (11, 12, 16, 17). A pooled analysis suggested that patients with pathological complete response after neoadjuvant chemoradiotherapy were less likely to benefit from adjuvant chemotherapy, whereas patients with residual tumor had superior outcomes when adjuvant chemotherapy was administered (13). In current study, from a large propensity-matched, population-based cohort, our results suggested significant overall survival benefits of adjuvant chemotherapy compared with observation for patients with rectal cancer receiving neoadjuvant radiotherapy and surgical resection. In addition, there is one of specific findings in our analysis that patients receiving neoadjuvant radiotherapy survive equivalently with stage-matched patients proceeded directly to surgical resection. This finding supports the accepted oncologic efficacy of neoadjuvant radiotherapy and the clinical application of neoadjuvant radiotherapy, as recommended by current guidelines (3, 21).

The role of adjuvant chemotherapy in management of rectal cancer has been established well. A Cochrane meta-analysis has shown a significant benefit in terms of disease-free survival (HR = 0.75, 95% CI 0.68–0.83) and OS (HR = 0.83, 95% CI 0.76–0.91) for patients with rectal cancer who received postoperative chemotherapy when compared with those undergoing observation alone (22). The phenomenon that adjuvant chemotherapy makes great effects on survival outcomes of patients with rectal cancer is also reflected in our exploratory analyses. The initial analyses in current study, regardless of adjuvant chemotherapy, showed significant improved survivals for patients with neoadjuvant radiotherapy than those without neoadjuvant therapy in stage II-III. It seems that patients yield superior benefit from neoadjuvant radiotherapy than stage-matched patients without neoadjuvant radiotherapy. Actually, the proportions of adjuvant chemotherapy in two groups are quite different, and the bias may be introduced by the effect of chemotherapy. Thus, our subsequent analyses in which patients were stratified by chemotherapy and draw an adjusted conclusion in which equivalence instead of superiority is suggested, as demonstrated above.

Since neoadjuvant chemotherapy are failed collected in SEER database, the present study analyzed patients receiving neoadjuvant radiotherapy, instead of neoadjuvant concomitant chemoradiotherapy. According to ESMO clinical practice guidelines, both two schedules of preoperative therapy, i.e. preoperative radiotherapy and chemoradiotherapy, are standards of care of rectal cancer (21). It is not possible to give a rigid definition of T and N sub-stages require short-course neoadjuvant radiotherapy or chemoradiotherapy; the selection of preoperative approach in locally advanced rectal cancer is based more regarding the risk of a positive circumferential resection margin at total mesorectal excision. If circumferential resection margin and/or R0 resection status are predicted at risk, neoadjuvant concomitant chemoradiotherapy is advised (23). Otherwise, either neoadjuvant short-course neoadjuvant radiotherapy or chemoradiotherapy can be administrated, offering similar oncologic outcomes and recurrence rate (24, 25). Although impact of concomitant chemotherapy was beyond our datasets, our analysis provide evidence to support the application of neoadjuvant radiotherapy for clinical management of rectal cancer (26).

Recently, there have been growing advances in development of survival prediction models and prognostication tools (27). A number of risk and outcome prediction models has been explored today for many important cancer types, such as gastric cancer (28, 29), gallbladder cancer (30), breast cancer (31, 32), and other sites. Although prediction model can never substitute for evidence from large-scale prospective randomized clinical trials, these tools are particular helpful to provide information in clinical decision-making in case of tumors for which rare data from clinical trials are available. Currently, application of neoadjuvant radiotherapy as well as the regimen and course duration, and clinical management strategy after neoadjuvant therapy are being explored and high-level evidence remains awaiting. In this study, we developed a model of nomograms to estimate survival benefit associated with neoadjuvant radiotherapy and adjuvant chemotherapy for rectal cancer. By considering those important clinicopathologic factors, the model estimates survival probability for patients with specific characteristics and therapeutic strategy, which could useful for decision-making in clinical setting.

This study has several limitations. First, although the patients were matched based on baseline characteristics in an attempt to minimize bias, unknown confounders not captured in the data set might produce residual bias in the results. Second, although the SEER is validated with quality insurance and training, its retrospective nature exposes the series to coding errors. This analysis is unable to identify reasons for why patients may not receive radiotherapy or chemotherapy such as patient fitness, or center related reasons. Third, the large, medical unit maintained, retrospective database affords little control over local practices and makes present analysis limit to available variables in the database. As such, duration and regimen of neoadjuvant radiotherapy and adjuvant chemotherapy, and surgical procedures are grouped broadly, and we are unable to evaluate and adjust for specific techniques, doses, or regimens. In addition, the absence of pretreatment clinical stage data limits our ability to understand effects of the degree of tumor downstaging by neoadjuvant radiotherapy on survival outcomes. This may rely on more full data from an expected prospective study to address.

## Conclusion

In summary, our findings suggested that patients with rectal cancer underwent neoadjuvant radiotherapy yield acceptable outcomes and are more likely to benefit from adjuvant chemotherapy in terms of overall survival. These data would be evidential for advocating consistency in guideline adherence to the use of adjuvant chemotherapy after neoadjuvant radiotherapy.

## Data Availability Statement

Publicly available datasets were analyzed in this study. This data can be found here: https://seer.cancer.gov/.

## Ethics Statement

Ethical review and approval was not required for the study on human participants in accordance with the local legislation and institutional requirements. Written informed consent for participation was not required for this study in accordance with the national legislation and the institutional requirements.

## Author Contributions

HW and XL guarantee for the integrity of the manuscript and contributed to the concept and design. ZC, SL, YW, ZF and NL contributed to analysis and interpretation of the data and the drafting and revision of the manuscript. All authors contributed to data collection, and editing, and revision of the manuscript. All authors contributed to the article and approved the submitted version.

## Conflict of Interest

The authors declare that the research was conducted in the absence of any commercial or financial relationships that could be construed as a potential conflict of interest.
